# Small Molecule Analogues of the parasitic worm product ES-62 interact with the TIR domain of MyD88 to inhibit pro-inflammatory signalling

**DOI:** 10.1038/s41598-018-20388-z

**Published:** 2018-02-01

**Authors:** Colin J. Suckling, Shahabuddin Alam, Mark A. Olson, Kamal U. Saikh, Margaret M. Harnett, William Harnett

**Affiliations:** 1 WestCHEM Research School, Department of Pure & Applied Chemistry, University of Strathclyde, Glasgow, UK; 2 Department of Immunology, Molecular and Translational Sciences Division, Army Medical Research Institute of Infectious Diseases, Frederick, MD 21702 USA; 3 Department of Cell Biology and Biochemistry, Molecular and Translational Sciences Division, Army Medical Research Institute of Infectious Diseases, Frederick, MD 21702 USA; 4 Institute of Infection, Immunity and Inflammation, University of Glasgow, Glasgow, G12 8TA UK; 5 Strathclyde Institute of Pharmacy and Biomedical Sciences, University of Strathclyde, Glasgow, G4 0RE UK

## Abstract

ES-62 is a protein secreted by the parasitic worm *Acanthocheilonema viteae* that is anti-inflammatory by virtue of covalently attached phosphorylcholine. Previously we have reported that drug-like Small Molecule Analogues (SMAs) of its phosphorylcholine moiety can mimic ES-62 in protecting against disease development in certain mouse models of autoimmune and allergic conditions, due to them causing partial degradation of the TLR/IL-1R adaptor MyD88. We have now taken a molecular modelling approach to investigating the mechanism underlying this effect and this predicts that the SMAs interact directly with the MyD88 TIR domain. Further support for this is provided by assay of LPS-induced MyD88/NF-κB-driven secreted alkaline phosphatase (SEAP) reporter activity in commercially-available stably transfected (TLR4-MD2-NF-κB-SEAP) HEK293 cells, as SMA**12b**-mediated inhibition of such SEAP activity is blocked by its pre-incubation with recombinant MyD88-TIR domain. Direct binding of SMA**12b** to the TIR domain is also shown to inhibit homo-dimerization of the adaptor, an event that can explain the observed degradation of the adaptor and inhibition of subsequent downstream signalling. Thus, these new data identify initial events by which drug-like ES-62 SMAs, which we also demonstrate are able to inhibit cytokine production by human cells, homeostatically maintain “safe” levels of MyD88 signalling.

## Introduction

**Figure 1:**
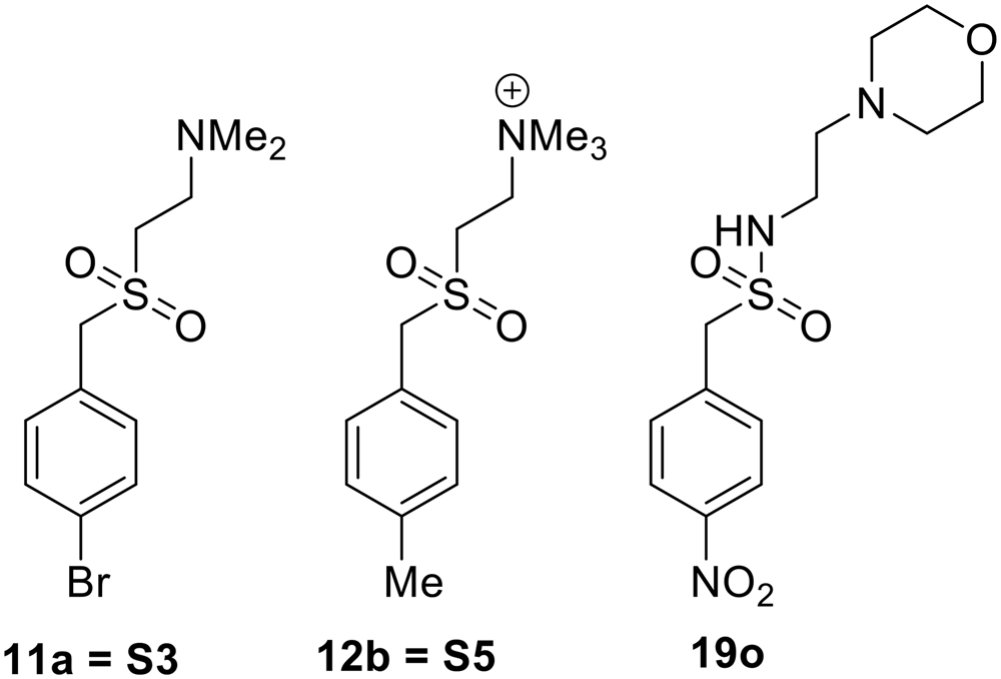
SMAs of ES-62 investigated in this work.

ES-62, a secreted product of the parasitic filarial nematode, *Acanthocheilonema viteae*^1^, has been shown in a number of studies to be a highly effective immunomodulatory agent. Specifically, this molecule can interfere with the pro-inflammatory responses of various immune system cells including B cells, dendritic cells (DCs), macrophages and mast cells. [reviewed^2^]. Such immunomodulatory properties dictate that ES-62 has protective effects in a number of mouse models of inflammatory disease, in particular collagen-induced arthritis (CIA)^3–6^, ovalbumin-induced airway-hyper-responsiveness (OAH)^7–9^, oxazolone-induced skin hypersensitivity (OSH)^7^, the MRL/Lpr model of systemic lupus erythematosus (SLE)^10^ and the *Gld*.ApoE^−/−^ model of accelerated atherosclerosis in SLE^11^. In spite of this, ES-62 *per se* does not directly have potential as a therapy, being a protein whose biological activity is dependent on post-translational attachment of phosphorylcholine moieties to an *N*–type glycan by a mechanism that is not fully characterised [reviewed^12^]. For this reason, a library of small molecule analogues (SMAs) based upon the active phosphorylcholine moiety but containing chemically stable substitutes for the phosphate ester, principally sulfonamides and sulfones, was designed, and evaluated by measuring ability to modify the cytokine output profile of stimulated macrophages^13^, DCs^14^ and mast cells^15^. Some of the sulfones were found to impact on cytokine output profiles in a manner similar to that of ES-62 and two in particular, **11a** (also known as S3) and **12b** (also known as S5)^13^, were selected for further evaluation. These have since been shown to be safe and effective when tested in CIA^13,16^, OAH^15^ and other airway hypersensitivity models employing clinically relevant allergens^17^, the MRL/Lpr mouse model of SLE^18^ and OSH^19^. Of note, the two SMAs acted at low doses (50 µg/kg), showed both prophylactic and therapeutic effects, and have recently been found to be protective in CIA when administered as DC therapy^14^. By contrast, the sulfonamides generally did not mimic the immunomodulatory effects of ES-62. Indeed, a typical example, **19o**, which was previously shown to have negligible effects on pro-inflammatory cytokine release from macrophages^13^ and not to protect against disease development in the MRL/Lpr SLE model^18^, was investigated further in the current study as a potential negative control. The structures of the 3 SMAs are shown in Fig. 1.

**Figure 2:**
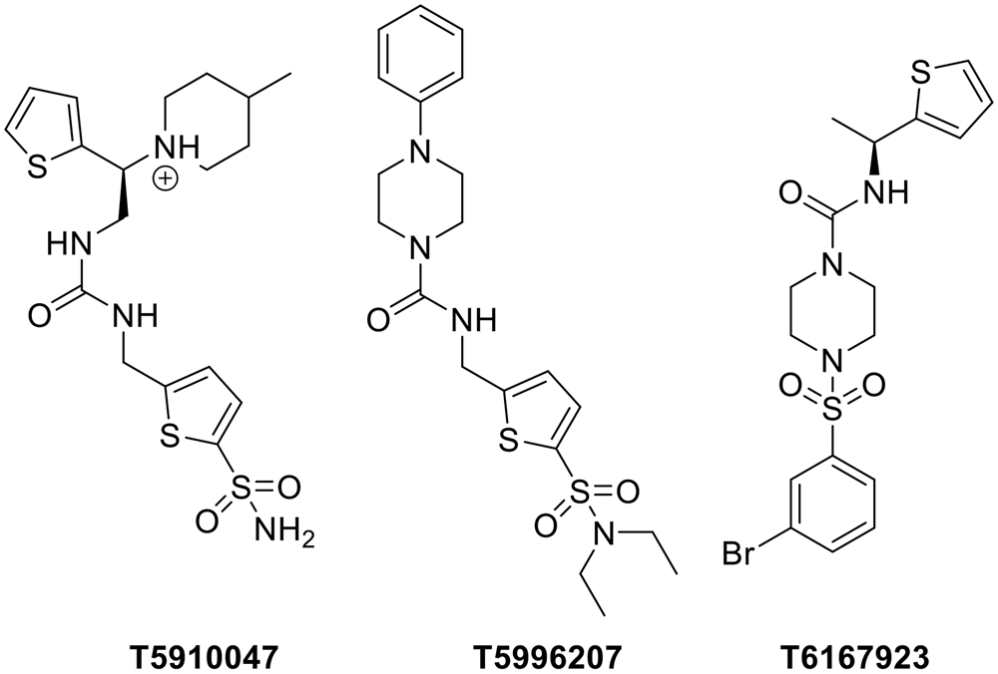
Compounds derived from *in silico* screening using the MyD88 dimerisation model^33^.

Although there may be additional targets for the SMAs, it is likely that the primary target, as with ES-62, is MyD88^4,10,20^, a 31–33 kDa adaptor protein consisting of a so-called N-terminal death domain and a C-terminal Toll/interleukin-1 receptor (TLR/IL-1R; TIR) domain separated by a short linker region, which acts as an anchor, recruiting signal transducers to TLRs/IL-1R and also other receptors including that for IFN-γ^21–26^. Consistent with this, both **11a** and **12b** reduce levels of this important adaptor molecule in bone marrow-derived macrophages^13,16^ and also kidney cells^18^. Imbalance of MyD88 function in the immune system can result in excessive stimulation of inflammatory signalling, leading to a wide range of syndromes and diseases, including those that ES-62 has been shown able to modulate in model systems. Because of its important role in signal transduction, MyD88 and its complexes have been identified as potential drug targets^27,28^ particularly, in reducing the inflammatory impact of sepsis following bacterial infection^29–31^, autoimmune connective tissue diseases^32^, and lymphomas^27^. In addition to peptide inhibitors of MyD88 dimerisation, small molecule inhibitors (“T-series” inhibitors) have been investigated using virtual screening of a compound library containing 5 million members via binding to a protein-protein dimeric docking model of the TIR-domain of MyD88^33^. From a first generation hit compound (T5910047; Fig. 2 LHS), others were identified from the PubMed database with more drug like properties (Centre and RHS; Fig. 2) and the most active compound (T6167923; RHS Fig. 2) evaluated in a range of immunological and biochemical assays relevant to MyD88 function, which indicated that it exhibited its anti-inflammatory actions via inhibition of MyD88 homodimerisation^33^. Compound T6167923 was also found to have therapeutic efficacy in protecting mice from staphylococcal enterotoxin B (SEB)-induced septic shock following administration of a single dose^33^. The similarity of these T-series compounds in terms of immunological profile (reducing the release of pro-inflammatory cytokines from stimulated cells) with that of the SMAs of ES-62 together with a number of common structural features with the SMA library suggested that it was possible that the active SMAs, **11a** and **12b**, by binding to the same or nearby sites on the MyD88 surface, might also disrupt its dimerisation. The ‘active’ SMAs, **11a** and **12b**, and the ‘inactive’ SMA **19o** were therefore examined initially as ligands for MyD88 *in silico*. Subsequently their effects on MyD88 protein complex formation and its downstream consequences were evaluated.

## Results

### Molecular modelling reveals potential binding sites for ES-62 SMAs in the MyD88 TIR domain

**Figure 3:**
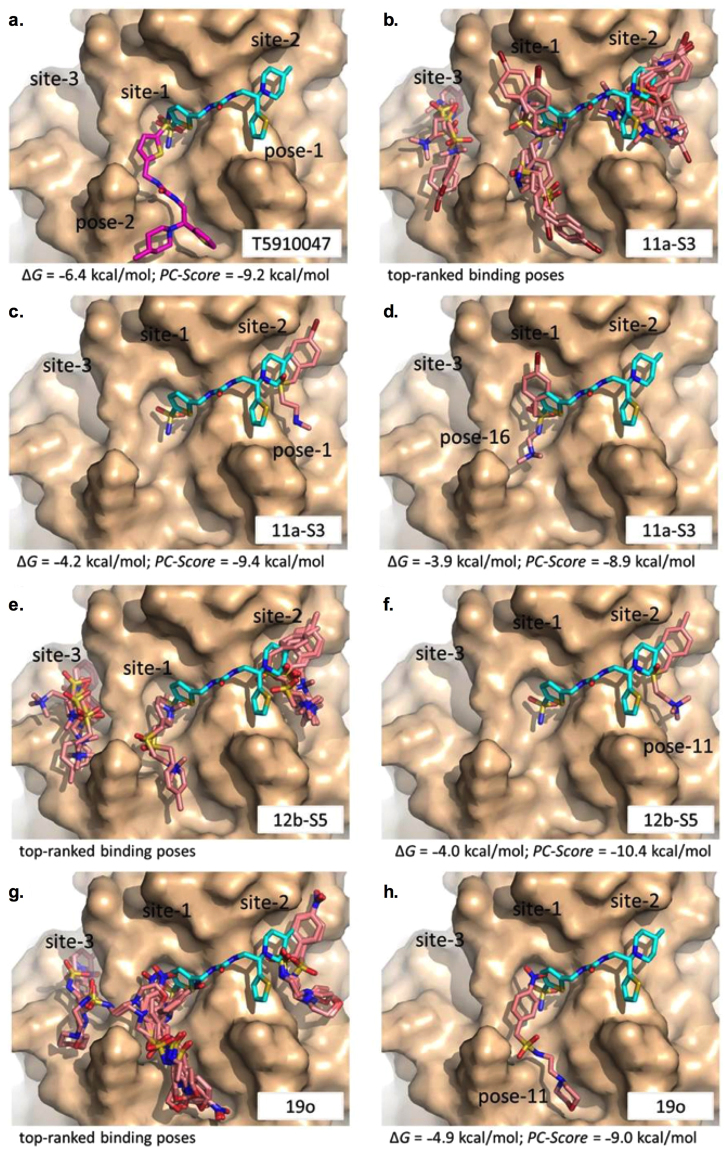
Molecular docking results for T5910047 and SMAs **11a**, **12b** and **19o**. The protein target MyD88 is illustrated as a molecular surface (coloured tan) with distinct binding regions annotated as site 1, site 2 and site 3. Docked molecules are illustrated as sticks with oxygen atoms coloured red, nitrogen coloured blue and sulphur coloured yellow. (**a**) Two distinct binding poses for T5910047 where binding to site-1 is coloured magenta and to site-2 is coloured cyan; (**b–d**) molecular docking of **11a** (coloured pink); (**e**,**f**) Docking of **12b** (coloured pink); and (**g**,**h**) docking of **19o** (coloured pink). Docking scores are given by Vina Δ*G* and *PC-Score* (see text).

The similarity between the SMAs and the T-series compounds was first evaluated formally using the previously described molecular modelling methods^33^. As a reference, Fig. 3a shows the docking of molecule T5910047 in two different binding poses and the overall top-ranked scores from Vina Δ*G* and the computed *PC-Score*. Because T5910047 was derived from *in silico* screening of roughly 5 million compounds without ligand-binding optimization or refinement and showed an inhibition level as a minimal threshold for compound selection, the T5910047 score is used as a benchmark for assessing the three ES-62 SMA compounds. The two binding poses of T5910047 illustrated in Fig. 3a are nearly indistinguishable in terms of scoring and are given by Vina Δ*G* = −6.4 kcal/mol and *PC-Score* = −9.2 kcal/mol. When enrichment of compound selection is taken into consideration based on T5910047^33^, the top-ranked 2nd-generation compound T6167923 (docking pose not shown) yielded a Δ*G* = −5.5 kcal/mol and *PC-Score* = −9.8 kcal/mol. Concurrence in predicted binding regions for T5910047 and T6167923 is observed at site-1 and site-2 of MyD88, while site-3 is less favourable in terms of docking populations among the generated ensemble.

While in general docking scoring functions are imperfect in detecting optimal conformational poses, the scoring method of *PC-Score* appears to offer the better guidance on ranking potential interactions for small molecules with MyD88. This is buttressed by the negligible statistical variance in *PC-Score* values among the top-ranked 25 binding poses for a selected molecule and as such, the differences in aggregate values can be applied to distinguish compounds. For the three SMAs, docking successfully sampled favourable binding modes on the MyD88 model, although unlike T5910047 and T6167923, docking populated the three binding sites (Fig. 3b,e and g). There were some similarities observed at functional group level between the SMAs and T-series compounds. Figure 3d shows the docking of **11a** in a binding pose where the sulfone functional group is recognized by the same binding pocket (site-1) as T5910047. The *PC-Score* = −8.9 kcal/mol and is slightly less favorable than that for T5910047 (−9.2 kcal/mol).

Of the three SMAs, **12b** scored most favourably in *PC-Score* (−10.4 kcal/mol), even performing better than T5910047 and T6167923. The docking of **12b** is shown in Fig. 3e & f. As with **11a**, this compound favoured binding to site-2, to which T5910047 binds in the model, but did not mimic the binding mode of T5910047 to that site. However, an alternative binding pose of **12b** to site-1 bound almost as strongly with *PC-Score* −10.2 kcal/mol and blocked the small pocket recognized by T5910047 in site-1 (Fig. 3e). The importance of this pocket as a possible recognition point for inhibitors reflects its peripheral location to the BB-loop region of MyD88, which is a conserved region in the TIR domain. In contrast to SMAs **11a** and **12b**, the best binding pose of SMA **19o** had a less effective *PC-Score* of −9.0 kcal/mol and performed similarly to T5910047. However, docking suggests that **19o** bound to site-1 in the model but in an orientation substantially different from that of T5910047 (Fig. 3g & h).

Together the docking results indicate that it is possible that the SMAs **11a** and **12b** might interfere with MyD88 function in a manner similar to T5910047 but that SMA **19o** might behave significantly differently; this is consistent with the inactivity of **19o** in cytokine stimulation profile experiments^13,16,18^. Further experimental evaluation of the actions of **11a** and **12b** on MyD88 signalling was therefore undertaken.

### ES-62 SMAs inhibit MyD88-dependent cell signalling and cytokine production

**Figure 4:**
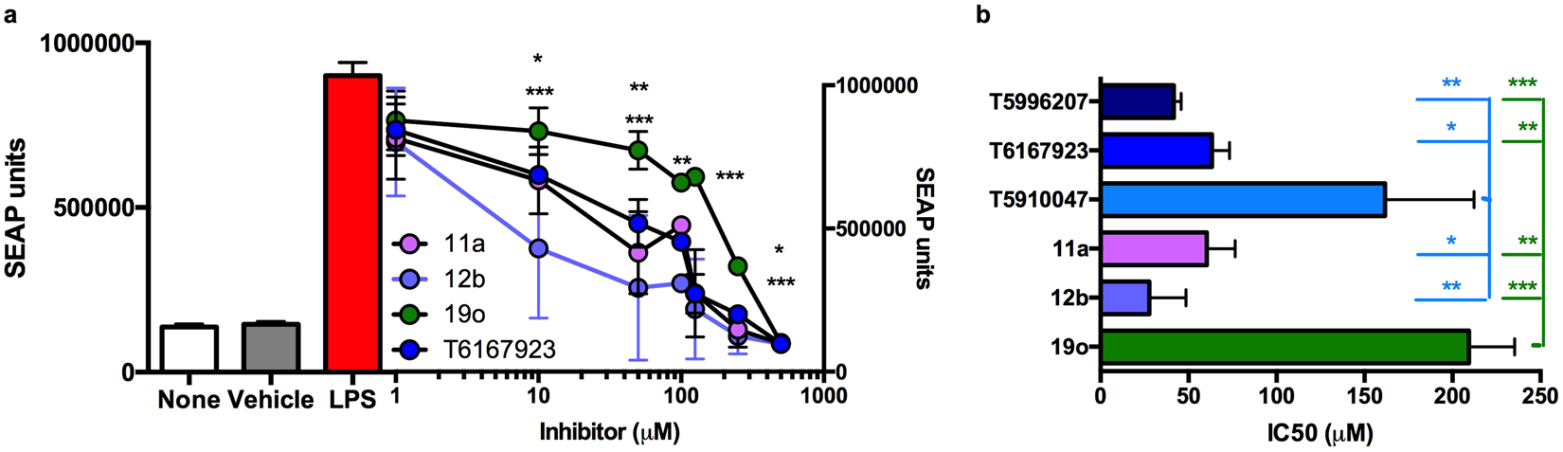
Inhibition of LPS-induced MyD88-mediated SEAP response by ES-62-SMAs. SMAs of ES-62 were tested in parallel with the indicated MyD88 T-series inhibitors^33^ for their effects on MyD88-signalling by monitoring LPS-induced, NF-κB-dependent SEAP activity. Briefly, the stably-transfected HEK 293 T cell line (TLR4-MD2-NF-κB-SEAP) was stimulated using LPS and treated with various concentrations of the indicated compounds (1–250 µM). (**a**) SEAP activity was determined in culture supernatants and data are presented as the mean ± SD values collated from 3 independent experiments, n = 9 unless otherwise indicated. Statistical analysis was by 2-way ANOVA with Bonferroni’s multiple comparison post-test showing that at: 10 µM, *p < 0.05 for T6167923 and **11a** v **19o**, ***p < 0.001 for T6167923 v **12b**, **11a** v **12b** and **12b** v **19o**; 50 µM (n = 6), **p < 0.01 for T6167923 v **12b** and **19o** and ***p < 0.001 for **11a** and **12b** v **19o**; 100 µM (n = 3), **p < 0.01 for **12b** v **19o**; 125 µM (n = 6), ***p < 0.001 for each of **11a**, **12b** and T6167923 v **19o** and for 250 µM, *p < 0.05 for T6167923 v **19o** and ***p < 0.001 for **11a** and **12b** v **19o**. (**b**) IC_50_ values were obtained using program Origin pro 7.5^30,33^ and represent the concentration required for inhibition of SEAP activity by 50% relative to the control. Again, data are the mean values ± SEM from three independent experiments, where *p < 0.05, **p < 0.01 and ***p < 0.001 for the various compounds versus T5910047 (blue) or **19o** (green) as indicated.

The effect of the SMAs in comparison with the T-series compounds on LPS-induced TLR4-dependent MyD88 signalling was investigated first using a cell-based reporter assay (SEAP) using protocols we described previously^29,30,33^. A stably co-transfected HEK 293 T cell line (TLR4-MD2-NF-κB/ SEAP) was employed to measure ligand (LPS)-induced MyD88-mediated NF-κB driven SEAP reporter activity (Fig. 4). Both of the compounds **11a** and **12b** inhibited LPS-induced MyD88–mediated SEAP expression in a dose-dependent manner, while, consistent with previous functional studies^13,16,18^ and potentially reflecting the modelling data (Fig. 3), **19o** showed very weak inhibitory action apart from at high concentrations. SMAs **11a** and **12b** were active between 1–10 μM, consistent with our previous findings demonstrating their inhibition of TLR4-driven functional responses (IL-6 release) in mouse bone marrow-derived macrophages at >3 μM^13^. These current findings were supported by analysis of the IC_50_ values (60+/−16 μM and 28+/−21 μM, respectively) obtained from 3 independent experiments that showed the potency of **11a** and **12b** to be not significantly different from the “best” second generation compound of the T-series, T6167923^33^ (63+/−10 μM) but stronger than that of the prototypic T5910047 compound, which perhaps consistent with their similar *PC-scores*, was not significantly different to the “inactive” ES-62 SMA, **19o** (Fig. 4).

**Figure 5:**
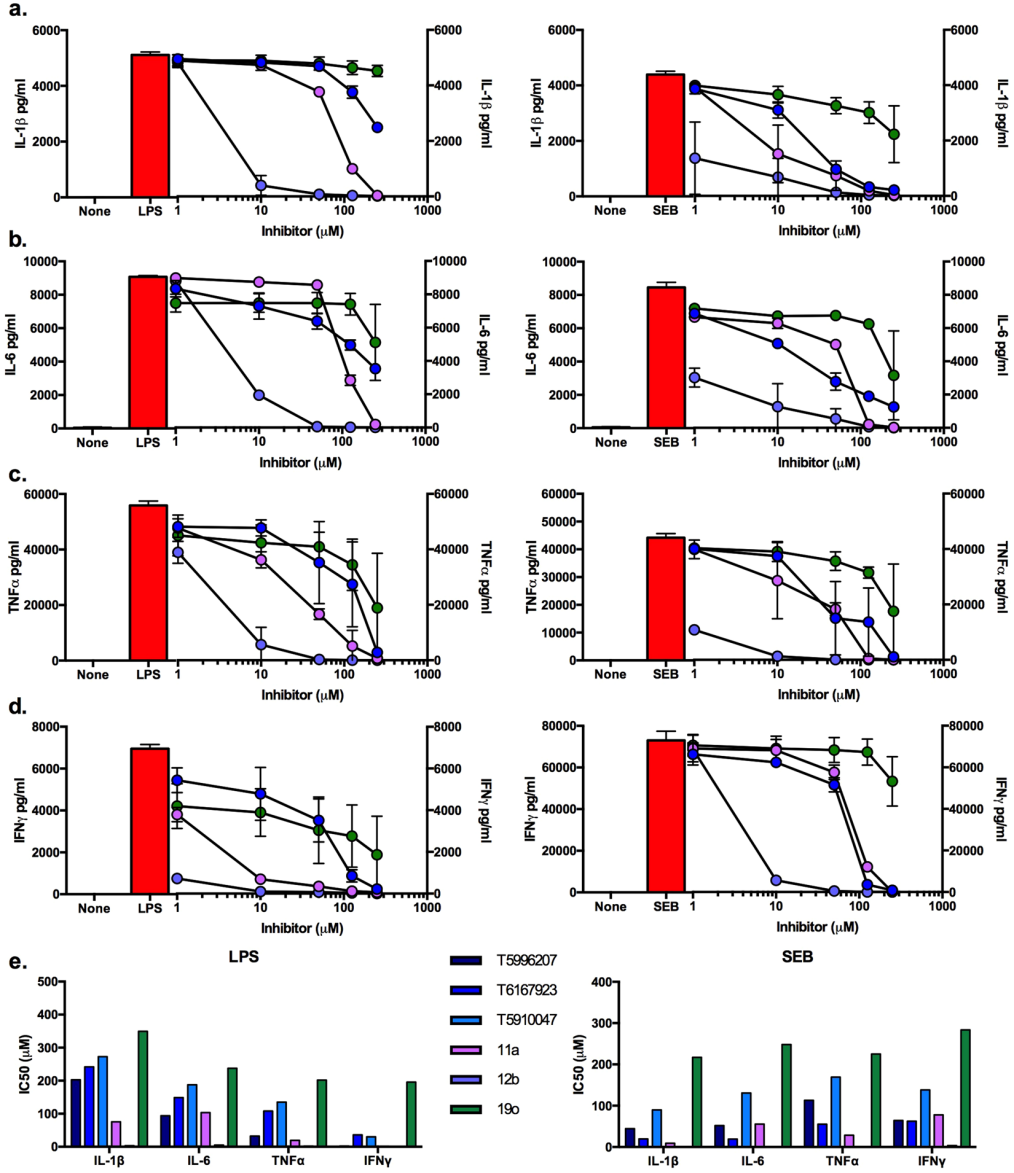
Inhibition of LPS- or SEB-stimulated cytokine production of human PBMCs by SMAs of ES-62. PBMCs (1 × 10^6^) from normal donors were cultured for 20 h with LPS (1 µg/ml; left panels) or SEB (200 ng/ml; right panels) with or without the indicated inhibitors (1–250 µM). The culture supernatants were collected and measured (in duplicate) for IL-1β (**a**), IL-6 (b), TNFα (**c**) and IFNγ(d) production by MSD assay^42–44^ and the data are presented as the means ±SD of duplicate samples (n = 4) from two independent experiments. Differential efficacy amongst the compounds was revealed by 2-way ANOVA, which for clarity is shown here (*p < 0.05, **p < 0.01 and ***p < 0.001) without the dose-by dose post-test analysis: thus, for IL-1β, all treatments were significantly different from each other at *** for both LPS- and SEB-stimulated responses; IL-6: LPS, T5910047 vs **11a** *, T5910047 vs **19o** **, T5910047 vs **12b** and **11a** vs**12b**/**19o** and **12b** vs **19o** all ***; SEB, T5910047 was not significantly different from **11a** but all other comparisons were ***; TNFα: LPS, apart from T5910047 vs **19o**, which was non-significant, all other comparisons were significant at ***; SEB, apart from T5910047 vs **11a**, which was non-significant, all other comparisons were significant at ***; IFNγ apart from LPS,T5910047 v **19o**, all comparisons were significant at ***. IC_50_ values (**e**) were determined as the concentration of compound required to induce 50% inhibition of cytokine production relative to the control cells from the plotting of dose response curves using a scatter plot followed by a sigmoidal curve fit.

Next, we assessed the effect of **11a** and **12b**, relative to the T series compounds, on LPS-induced MyD88-driven pro-inflammatory cytokine signalling in primary human peripheral blood mononuclear cells (PBMCs) (Fig. 5, left-hand panels). This showed **12b**, in particular, to be substantially more potent than T6167923, in inhibiting LPS-induced release of IL-1β and IL-6 from PBMCs (Fig. 5a & b). Moreover, both **11a** and **12b** were considerably more effective than T6167923 in inhibiting release of TNFα and IFNγ from such cultures (Fig. 5c & d) and indeed, even **19o** proved somewhat active against the release of IFNγ: these perhaps unexpected IFNγ data may reflect that release of this cytokine can result from both indirect (via IL-12 production) and direct (on NK cells) actions of LPS on PBMCs^34,35^, with the latter possibly explaining the potent activity of all 3 SMAs (1 µM) against IFNγ production. These data are reinforced by the IC_50_ values determined for the ES-62 SMAs and T series compounds (Fig. 5e), where similarly to the SEAP activity, cytokine inhibition was achieved at a lower concentration of **12b** than of **11a**, and also relative to the T-series compounds. These results support the proposed MyD88 target specificity of the compounds **11a** and **12b** in inhibiting NF-κB driven SEAP activity and cytokine release.

Other receptors besides TLRs signal via MyD88, for example IFNγ and MHC-mediated responses also engage this adaptor molecule^21–26^. Indeed, our previous results demonstrated that SEB activates MyD88-mediated pro-inflammatory cytokine signalling via binding to MHC class II molecules on primary human monocytes^36^ and consistent with this, there is a decrease in serum levels of such cytokines induced by exposure to SEB in MyD88 gene knockout (MyD88^−/−^) mice^37,38^. It should also be borne in mind, however, that SEB may impact on PBMCs via TLR2-MyD88 signalling^39,40^. Nevertheless, to determine whether by targeting MyD88, **11a** and **12b** would also be capable of inhibiting SEB induced pro-inflammatory cytokine responses, we stimulated primary human PBMCs with SEB in the presence of the SMAs (Fig. 5, right-hand panels). Dose response analysis again showed **12b** to be the most potent compound by a substantial margin, whilst **11a** and T6167923 generally displayed similar activities. Also, for this stimulus, **19o** was the least active compound tested and in contrast to what was observed for the LPS cultures, did not show activity against the (much stronger) IFNγ responses elicited by SEB (Fig. 5a–d). Moreover, the IC_50_ values (Fig. 5e) showed the relative potencies, apart from those relating to the IFNγ responses, to be broadly consistent with the data obtained with LPS stimulation. Collectively, as we have previously shown for TLR-MyD88 signalling in mouse BMMs^13^, these data potentially suggest some selectivity amongst the SMAs with respect to the receptor-MyD88 coupling targeted.

### SMA 12b interacts directly with MyD88 to inhibit its dimerisation and results in MyD88 downregulation

**Figure 6:**
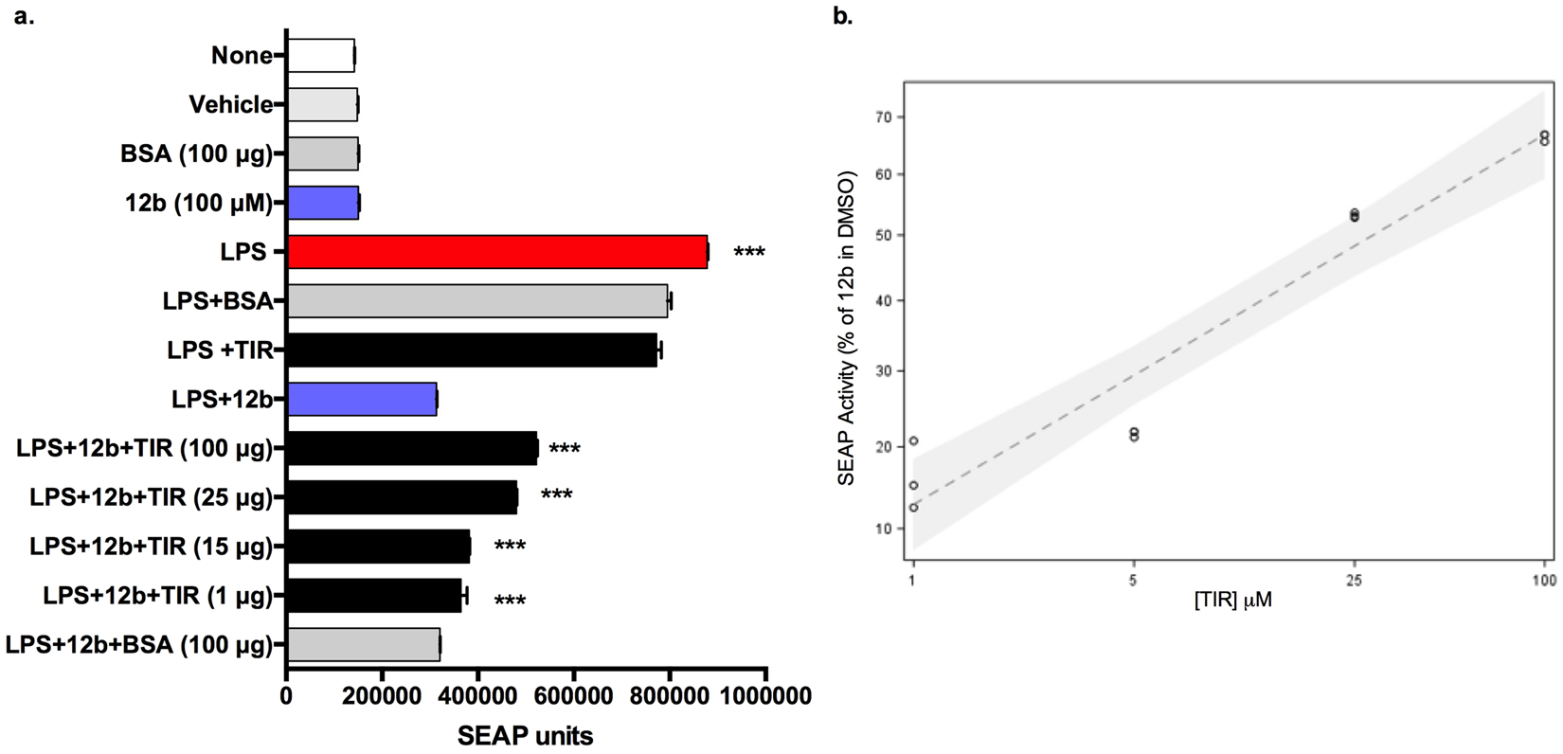
MyD88 TIR-domain validation of the target specificity of compound **12b**. To determine whether SMA **12b** inhibits MyD88-signaling by direct binding to the TIR domain, the SMA was pre-incubated at room temperature with varying amounts of TIR domain protein (or with BSA as control) for 2 h prior to being added to cells for testing for SEAP activity as described in Fig. 4. Data (**a**) are presented as mean ±SD SEAP response units from triplicate analysis of a single experiment where *p < 0.05, **p < 0.01 and ***p < 0.001 for conditions of interest versus LPS + **12b** by 1-way ANOVA and Bonferroni post-test and (**b**) The log-log linear regression model is fitted to the data relating SEAP activity to TIR concentration as shown in equation 1 below by analysis carried out in SAS version 9.4 SAS proc MIXED. *Equation*: $Log(\tfrac{SEAP\,Activity}{Activit\,of\,s5\,in\,DMSO})$ = $\beta \,Log([TIR])+\alpha$.

To confirm the SMAs functionally reduce MyD88 signalling by directly binding to the TIR domain of MyD88, we performed the LPS-induced cell-based SEAP reporter assay after pre-incubation of the more potent of the two active compounds in this study, SMA **12b** (100 µM), with different amounts of TIR domain protein (1–100 µg). Pre-incubation with TIR domain protein was found to block the inhibitory effect of **12b** on SEAP expression in a dose-dependent manner, as shown by a greater SEAP response when compared to that observed following pre-incubation with BSA as a control protein (Fig. 6a). These results clearly demonstrate the specificity of SMA **12b** in targeting the TIR domain of MyD88 and indicate that direct binding of the SMA to the TIR protein reduced the inhibitory effect of the compounds on MyD88-signaling. To gauge the quantitative impact of SMA action in binding to MyD88, analysis of the effects of 2 independent experiments showed that whilst exposure to **12b** resulted in an average of 77% inhibition of the LPS response, this was substantially reduced by 25 µg TIR (to 48% inhibition) but not BSA (74% inhibition, i.e., comparable to the 77% described above). Indeed, it was estimated that each 5-fold increase in TIR concentration produced a 14.64% increase in SEAP activity (95% confidence interval 11.647 to 17.720%), whereas essentially no change in activity was observed with BSA protein. Regression analysis shows this to be a statistically significant effect (*P* < 0.05; Fig. 6b).

**Figure 7:**
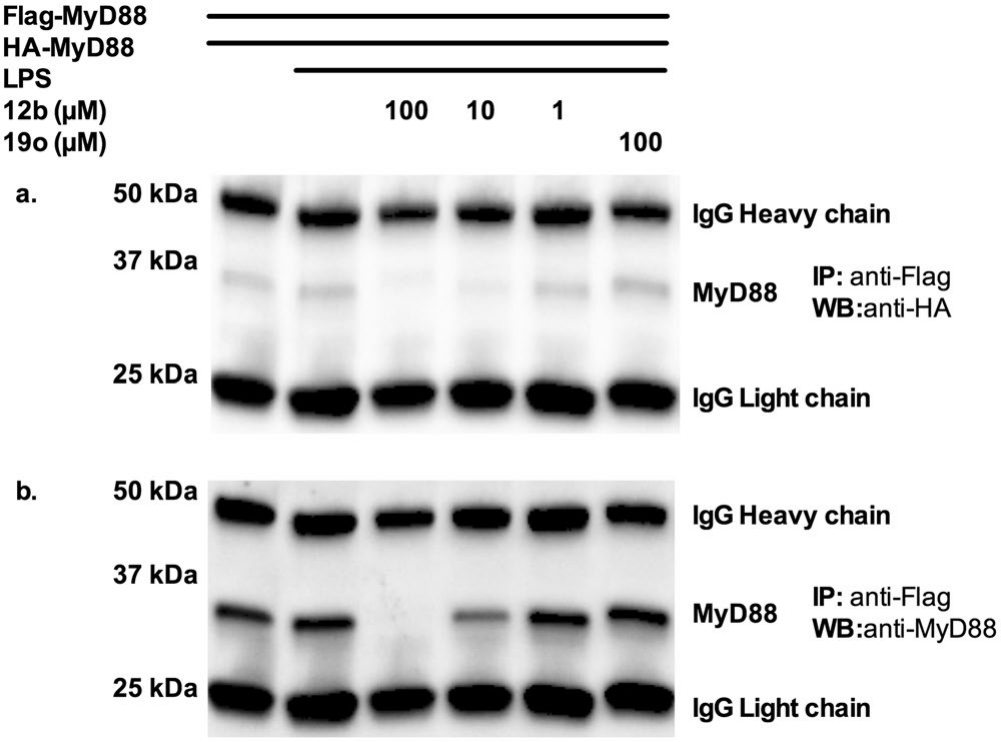
Inhibition of MyD88 homodimer formation in the presence of SMA **12b**. HEK 293 T cells (TLR4-MD2-NF-κB-SEAP) co-transfected with plasmids MyD88-Flag or pCMV-HA-MyD88 for 7 h were incubated for a further 13 h with LPS in the presence or absence of the compound **12b** (100 µM to 1 µM) or control compound **19o** (100 µM). Cell lysates were immunoprecipitated (IP) using an anti-Flag antibody, and the resultant immune-complexes analyzed by Western blotting (WB) employing an anti-HA antibody (**a**). The identity of the 31 kDa MyD88 band was validated by re-probing with an anti-MyD88 antibody (**b**).

Finally, as the dimeric form of MyD88 is generally required for signal transduction and pro-inflammatory cytokine responses^25,26^, we next determined whether SMA **12b** binding to the TIR domain of MyD88 is reflected by it preventing homodimerisation of newly synthesized MyD88 to inhibit LPS-induced MyD88 mediated signalling for pro-inflammatory responses. Specifically, we examined MyD88-deficient HEK 293 T cells that were co-transfected with plasmids pCMV-HA-MyD88 and MyD88-Flag and 7 h later incubated with LPS in the presence of various concentrations of compound **12b**. Cell lysates were co-immunoprecipitated using anti-Flag antibody, and then analysed by SDS-PAGE followed by immunoblotting, the latter employing an anti-HA antibody. In addition, after stripping of the blot, re-probing using anti-MyD88 antibody was undertaken to validate the identity of the 31 kDa band as MyD88. As we have shown previously, LPS binding to TLR4 induced a slightly higher level of HA-MyD88 and total MyD88 expression^29,31,38^ in the immune complexes. By contrast, in the presence of compound **12b**, the levels of newly expressed Flag-MyD88/HA-MyD88 homodimer were reduced, with maximum inhibition of HA-MyD88 expression being observed at between 10 µM and 100 µM. By contrast, no inhibition of MyD88 homodimerization in terms of HA-MyD88 expression was observed with control compound **19o** at the highest concentration (Fig. 7). Although the levels of total MyD88 detected were generally not so dramatically inhibited as those of HA-MyD88 (e.g. ~50% versus ~80% at 10 µM respectively) due to the anti-MyD88 antibody detecting the presence of monomeric Flag-MyD88 in the immune complexes, re-probing with anti-MyD88 antibody provided supporting evidence for exposure to compound **12b** resulting in inhibition of MyD88 homodimerisation (Fig. 7). In addition, this approach to analysing MyD88 dimerisation also indicated that a consequence of its disruption is downregulation of the adaptor molecule as evidenced by the almost complete loss of detection of newly synthesised MyD88 at the highest SMA concentration. By contrast but in agreement with our earlier reports demonstrating that **19o** does not downregulate MyD88 expression^13,16,18^, the levels of MyD88 expression were not reduced as indicated by the levels of total MyD88. These data demonstrating inhibition of Flag-MyD88 and HA-MyD88 dimerisation and associated downregulation by SMA **12b**, are consistent with the effects of this ES-62 SMA on SEAP reporter expression and cytokine inhibition (Figs 4 and 5). Certainly, inhibition of dimerization of the adaptor would be likely to result in reduction in downstream signalling as the dimeric form of MyD88 is generally required for signal transduction and pro-inflammatory cytokine responses^25,26^ whilst monomeric MyD88 is susceptible to degradation via autophagy^39^.

## Discussion

This study was undertaken to investigate the possibility that SMAs of the immunomodulatory protein, ES-62, might owe their activity at least in part to interacting directly with the scaffolding protein, MyD88 with consequent inhibition of its dimerisation resulting in our previously observed downregulation of MyD88 expression^13,16,18^. SMAs of ES-62, principally those designated **11a** and **12b** (Fig. 1) have been shown to be effective in modulating immune responses in cellular studies and *in vivo* models of disease, in the latter case with substantial quantifiable improvement in disease-related metrics^9,13–19^. The first implication of the involvement of MyD88 came from the observation that its levels were decreased on treatment with SMAs in studies of a mouse model of CIA^13,16^. Whilst the loss of TLR signalling resulting from downregulation of MyD88 was consistent with the observed reduction in cytokine output, there was no evidence from this previous work to suggest that the SMAs interacted directly with MyD88.

Other studies have however, aimed to design molecules that target MyD88 by direct interaction, and have shown for example that the dimerisation of MyD88 can be impeded by small, synthetic compounds, typically peptides that had been designed to bind to the surface of the TIR subunit of the adaptor molecule^27^. One study that was of particular interest involved the virtual screening of 5 million compounds through a molecular model of the TIR subunit interface that led to the identification of a significant number of active compounds, referred to in this paper as the T-series (Fig. 2)^33^. The molecular model built for the discovery of the T-series compounds was also appropriate for the evaluation of the SMAs. Three SMAs were chosen, **11a** and **12b**, which were known to be active in cell-based studies and *in vivo*, and **19o**, which exhibited virtually no activity, as a negative control. The docking studies carried out with the SMAs using the same protocol as for the discovery of the T-series showed that the two sets of compounds could indeed bind at the same sites as the T-series (Fig. 3). Of the many potential binding poses, quantitative evaluation of the most tightly binding showed that **11a** and **12b** bound to the same sites as the T-series compounds with similar potency. For **11a** some poses, although not the tightest binding, had the sulfone binding in the same small protein pocket as the sulfonamide in the T-series compound, T5910047 (Fig. 3d), which was encouraging but not necessarily highly significant due to the general inactivity of sulphonamide-based SMAs referred to earlier. On the other hand, inactive **19o** bound in a different orientation, although with similar calculated affinity. The molecular model therefore reproduced the behaviour expected from the known biological properties of the SMAs under investigation.

The two active SMAs, **11a** and **12b**, were taken forward in cell-based assays to confirm their effects on MyD88-dependent signalling as shown by NFκ-B driven SEAP activity and cytokine release profiles from human cells in comparison with the T-series compounds (Figs 4 and 5) and in both sets of experiments the SMAs were found to be as effective or better than the T-series compounds. It was also noted that the activity of **11a** and **12b** was similar to that of a previously described MyD88 dimerisation inhibitor known as 4210 that targets the BB-loop in the TIR domain of MyD88^30^. In the present study, an effect on the release of four principal pro-inflammatory cytokines, IFN-γ, IL-1β, IL-6, and TNF-α was observed with respect to the use of both SMAs. With LPS as the stimulator, both SMAs were more potent than the T-series compounds and with SEB exposure, inhibition was similar or greater than with the T-series compounds: **12b** was found to be particularly potent with low and submicromolar IC_50_ values observed.

The molecular modelling and cytokine production data raised the possibility that treatment of cells with SMA **11a** or **12b** might have a direct effect on MyD88 and clear evidence for the engagement of **12b** with MyD88 was obtained, by the inhibitory effect of added recombinant TIR domain on the SMA’s ability to inhibit SEAP activity in the reporter assay. Although **11a** was generally found to be 5–10 fold less potent than **12b** in the cytokine release assays (Fig. 5), it is reasonable to infer that overall it operates in a similar manner to **12b** in binding to the MyD88 TIR domain, bearing in mind the overall likeness of the two SMAs’ behaviour in the range of molecular modelling and cellular evaluations reported above.

Recruitment of MyD88 as a dimer is a prerequisite for MyD88-mediated downstream signalling for pro-inflammatory responses and consistent with this, a reduction in homodimerisation accompanied by downregulation of MyD88 expression was observed when adopting a co-precipitation/Western blotting approach with cells transfected with MyD88 carrying two distinct labels. Our data are consistent with a recent independent report that the cellular consequence of blocking MyD88 dimerisation is that it becomes vulnerable to autophagolysosomal degradation^39^ and now provide a mechanism for our previous observations that the protection against CIA afforded by SMAs **11a** and **12b** is associated with downregulation of MyD88^13,16^. Importantly, this mechanism also resonates with our recent studies demonstrating that ES-62 harnesses the homeostatic and selective autophagolysosomal degradation of MyD88 and other TLR adaptor/transducers like Traf6 and PKCδ to limit pro-inflammatory responses of dendritic cells^40,41^.

The identification of MyD88 as a target of the SMAs **11a** and **12b**, and by analogy other active compounds in the class^14^, is important for their potential development as therapeutic agents in inflammatory and autoimmune diseases. Certainly, our earlier studies with T-series inhibitors, as well as with a BB-loop mimetic, designed by a structure-based approach to target the MyD88 TIR domain and tested by *in vitro* SEAP based reporter assays^30,31,33,38^ demonstrated therapeutic efficacy against toxic shock-induced death when tested in a mouse model of SEB intoxication. Thus, the SEAP reporter used in this study in evaluating the functional relevance provides biological significance of the results of **11a** and **12b**. It is also worth emphasising that the *in vivo* effects of the SMAs are to modulate the relevant downstream signalling pathways and not to block them as a kinase inhibitor would. It could be argued that modulation is an inherently safer therapeutic strategy than gross inhibition since its aim is to restore the natural balance in the context of the relevant biological system. The lack of toxicity observed with the SMAs in the now substantial range of *in vivo* models studied^9,13,15–19^ is consistent with this principle. This in turn suggests that it would be prudent in cases of disease of immunological imbalance such as autoimmune conditions, as opposed to infectious diseases in which killing the infecting organism is the therapeutic aim, to accept a broader drug discovery paradigm than the traditional one of single compound-single target-single effect, a paradigm that is rarely met completely by drugs in the clinic in any case.

## Materials and Methods

### Reagents

ES-62 SMAs **11a**, **12b** and **19o** were prepared as described previously^13^. T910047, T6167923 and T5996207 were purchased from Enamine, Ltd (La Jolla, CA). *Staphylococcal* enterotoxin B (SEB) - endotoxin-free and prepared under GMP conditions -was obtained from Porton Down, Inc. (Salisbury, UK) and stored at −50 °C. *Escherichia coli* (ssp. 055:B5) lipopolysaccharide (LPS) was from Sigma-Aldrich (St. Louis, MO). The multi-spot array ultrasensitive cytokine assay kit was purchased from Meso Scale Discovery (MSD; Gaithersburg, MD). Ficoll-Hypaque was purchased from GE Healthcare Biosciences (Piscataway, NJ), pooled human AB sera were obtained from Pel-Freez (Brown Deer, WI) and the anti-MyD88 antibody was obtained from AnaSpec, Inc. (San Jose, CA). The recombinant MyD88 TIR domain (157–296) protein was expressed in *E*. *coli* and purified as described in our earlier report^33^. The stably-transfected HEK 293 TLR4-MD2-NF-kB-SEAPorter cell line was purchased from Imgenex (San Diego, CA) whilst plasmids 12287 (pCMV-HA-MyD88) and 13093 (MyD88-Flag) were obtained through an MTA agreement with Addgene (Cambridge, MA). The transfection reagent lipofectamine was from Invitrogen (Carlsbad, CA).

### Molecular modelling

The computational molecular docking methodology applied in this study is slightly different from earlier published work^33^, which was focused on large-scale screening of commercially-available compounds. Here, molecular docking simulations are applied to evaluate SMA compounds as potential binders to the protein MyD88. Molecular docking calculations were conducted by using the program AutoDock Vina^42^. The human MyD88 protein was taken from the NMR structure listed as 2Z5V in the PDB^43^ and modelled as a rigid-body target in docking. For each modeled compound, a docking dataset of 2000 binding poses to MyD88 were extracted from the simulation generating roughly 2 M sampled conformations. The modeling protocol of defining atomic charges and docking parameters was identical to our previous work^33^.

To help detect native-like binding poses from decoys and provide a relative ranking of compounds, two scoring functions were applied. The first scoring function is that built into the AutoDock Vina molecular docking program. The function (denoted as the free-energy potential difference Δ*G*) contains electrostatic interactions through the hydrophobic and the hydrogen bonding terms in addition to a solvation/desolvation contribution. The second scoring function applied is the knowledge-based function DSX^44^. Our implementation of DSX is an approach of computing a per-contact score (designated as *PC-Score*) and is formulated as weighted field energy divided by the number atom-atom interactions between a ligand and MyD88 (computed within a distance of 6 Å). In contrast to the Vina scoring function, *PC-Score* yields greater weight to protein-ligand interactions rather than the thermodynamic cycle of complex formation.

### Cell Assays

Peripheral blood mononuclear cells (PBMCs) were obtained by consent from healthy donors in accordance with Institutional Review Board (IRB)-approved research donor protocol FY05–05. The minimal risk phlebotomy protocol employed was carried out in accordance with the approved guidelines of Office of Human Use and Ethics (OHU & E). An informed written consent was obtained from participants and reviewed by the USAMRIID physician (USAMRIID Screening and Eligibility). PBMCs were isolated by standard Ficoll-Hypaque density gradient centrifugation and suspended in RPMI 1640 medium as described elsewhere^31^. All of the experimental protocols where PBMCs were used were approved by OHU&E and Human Use Committee.

### Cytokine analysis

Purified PBMCs were cultured with LPS (1 µg/ml) or SEB (200 ng/ml) in the presence of varying concentration of SMAs by incubation at (37 °C, 5% CO_2_) for 16 h. Culture supernatants were collected and measured for cytokines using Meso Scale Discovery (MSD) multi-spot array ultrasensitive cytokine assay kit according to the manufacturer’s protocol as described in our previous study^33^.

### Secreted alkaline phosphatase (SEAP) reporter assays

SEAPorter^TM^ HEK 293 cells (TLR4/MD-2/NF-κB/SEAP) were cultured (5 × 10^5^ cells/ml/well in 24 well plates) with LPS (1 µg/ml), in the absence or presence of the indicated concentrations of test compounds at 37 °C for 16 h. Following centrifugation to remove cell debris, the culture supernatants were collected and the Great EscAPe SEAP Assay from Clonetech was used to determine the amount of secreted alkaline phosphatase as we described previously^33^.

### Co-immunoprecipitation and Western blot analysis

MyD88-deficient HEK 293-I3A cells were co-transfected with plasmids pCMV-HA-MyD88 and MyD88-Flag and 7 h later, stimulated with LPS in the absence or presence of various concentrations of compounds **12b** or **19o** for 13 h. Following washing with ice-cold PBS, cells were lysed and cytosolic fractions collected for immunoprecipitation as described previously^33^. Cell extracts (1 mg total protein) were incubated for 16 h under conditions of continuous shaking at 4 °C with 2 µg of mouse anti–Flag M2 antibody conjugated to agarose attached to magnetic beads (Sigma-Aldrich). Following recovery using a magnetic separator, agarose bead-bound immunocomplexes were washed three times, eluted in SDS-PAGE sample buffer and then separated by SDS-PAGE prior to Western blot analysis^33^ with anti-HA antibody followed by stripping the gel and reprobing with anti-MyD88.

### Statistical analysis

As indicated, data were analysed by 2-way or 1-anova with Bonferroni’s post-test.

The log-log linear regression model fit was utilized for the data relating SEAP activity to TIR concentration as shown below in equation 1:

1 $$Log(\,\frac{SEAP\,Activity}{Activit\,of\,s5\,in\,DMSO}\,)=\beta \,Log(\,[TIR]\,)+\alpha $$

Analysis was carried out in SAS version 9.4 SAS proc MIXED.

## Electronic supplementary material

Supplementary Information
