# Supplementary material for: Small Molecule Analogues of the parasitic worm product ES-62 interact with the TIR domain of MyD88 to inhibit pro-inflammatory signalling
Source: Sci Rep. 2018 Feb 1;8:2123. doi: 10.1038/s41598-018-20388-z (PMC5794923; doi:10.1038/s41598-018-20388-z)

Small Molecule Analogues of the parasitic worm product ES-62 interact with the TIR domain of MyD88 to inhibit pro-inflammatory signalling

Colin J Suckling[1], Shahabuddin Alam[2], Mark A Olson[3], Kamal U Saikh[2], Margaret M Harnett[4*] and William Harnett [5*]

[1]WestCHEM Research School, Department of Pure & Applied Chemistry, University of Strathclyde, Glasgow, UK; [2]Department of Immunology, and [3]Department of Cell Biology and Biochemistry, Molecular and Translational Sciences Division, Army Medical Research Institute of Infectious Diseases, Frederick, MD 21702, USA; [4]Institute of Infection, Immunity and Inflammation, University of Glasgow, Glasgow G12 8TA, UK; [5]Strathclyde Institute of Pharmacy and Biomedical Sciences, University of Strathclyde, Glasgow G4 0RE, UK

*Joint corresponding authors

Margaret Harnett, Institute of Infection, Immunity and Inflammation, College of Medical, Veterinary and Life Sciences, University of Glasgow, Glasgow G12 8TA, UK; Phone – 0044-141-330-8413; e.mail – Margaret.Harnett@glasgow.ac.uk

William Harnett, Strathclyde Institute of Pharmacy and Biomedical Sciences, 161 Cathedral Street, University of Strathclyde, Glasgow G4 0RE, UK; Phone – 0044-141-548-3715; FAX: 0044-141-552-2562; e.mail: w.harnett@strath.ac.uk

Supplementary Figure 1 Representative full-length images of the Western Blot analysis of expression of MyD88 and HA-MyD88 (Fig. 7), shown with molecular weight standard markers.

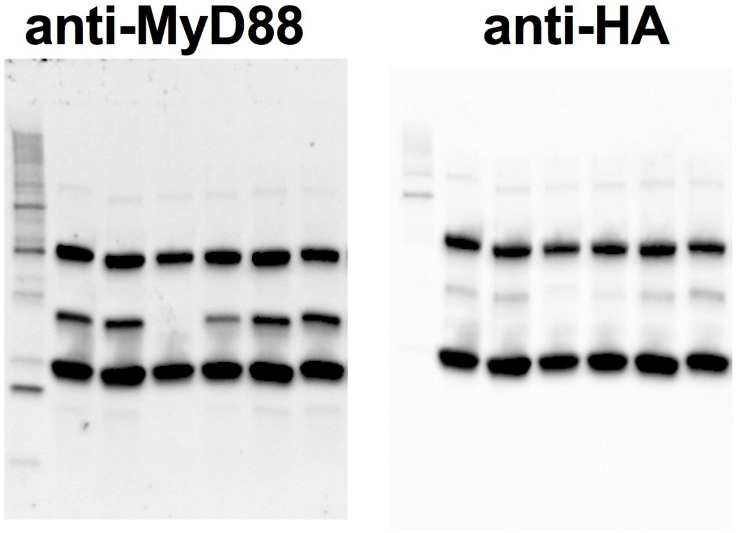

Supplement: Supplementary file 1 — Supplementary Information [file 41598_2018_20388_MOESM1_ESM.pdf]
